# Patterns of accidental deaths in Kuwait: a retrospective descriptive study from 2003–2009

**DOI:** 10.1186/s12889-015-1630-8

**Published:** 2015-03-28

**Authors:** Nadia Al-Kandary, Salah Al-Waheeb

**Affiliations:** Director of the Forensic Pathology Unit, General Department of Criminal Evidence, Ministry of Interior, Farwaniyah, Kuwait; Assistant Professor of Pathology, Faculty of Medicine, Kuwait University, Po Box 72, Shamiyah, Kuwait City, Code 71661 Kuwait

**Keywords:** Accident, Death, Asphyxia

## Abstract

**Background:**

Accidents are a preventable cause of death. Unfortunately it accounts for a large number of deaths in many societies. In Kuwait, road traffic accidents (RTA) is the leading cause of death in young people. The study investigated the patterns of accidental deaths in Kuwait, one of the Gulf States which incorporates a wide variety of multi-ethnic communities.

**Methods:**

The study was retrospective from 2003–2009. Data of forensic cases were collected from the general department of criminal evidence (GDCE) in the ministry of interior (MOI).We attempted to find out causes of accidental death and the prevelance of each cause. Furthermore, the relationship of demographic factors (eg. Age, sex, marital status and nationality) with each cause of accidental death in Kuwait were studied.

**Results:**

The material of this study constituted a total of 4886 reported accidental deaths referred for Medico-legal examination. Road traffic accidents was by far the most prevalent cause of death (64.6%) followed by fall from height (13.1%). Poisoning and mine explosions were amongst the least common causes.

**Conclusion:**

The government of Kuwait needs to take strong measures to promote safety in the workplace and households by educational campaigns.

## Background

Indirect estimates by the World Health Organization (WHO) and the Global Burden of Diseases Study (GBD) suggest that unintentional injuries account for 3.9 million deaths worldwide [1], of which about 90% occur in low- and middle-income countries. The majority of these deaths are attributable to road traffic injuries, falls, drowning, poisoning and burns [1]. Kuwait has a high incidence of RTA, however, it has also been noticed by the authors that accidental deaths in general have been reported commonly in the newspapers and in the media. Our objective in this paper is to review the types of accidental deaths in Kuwait over a seven year period (2003–2009). We would like to investigate the incidence of accidental deaths, the different types of accidental deaths that are prevalent in our country and how they relate to age, gender, nationality, marital status and governate. Ultimately the aim is to uncover the roots of the problems and attempt to find solutions to these problems.

## Methods

This descriptive study was conducted over the period of seven years from 2003 to 2009. This study involved all the accidental deaths in all the six Governorates in Kuwait. The data were collected from the GDCE in the MOI of the state of Kuwait.

The manner of death was classified as accident according to “A Guide for Manner of Death Classification” issued by the National Association of Medical Examiners [2]. An accident applied when an injury or poisoning causes death and there was little or no evidence that injury or poisoning occurred with the intent to harm or cause death. In essence, the fatal outcome was unintentional.

The material of this study constituted a total of 4886 cases reported as accidental deaths referred for Medico-legal examination during the period of January 2003 through December 2009. In order to identify the cause of death in each case, a full review of the data was made. This included epidemiological data, scene examination and radiographic investigations. Not all cases were subject to a full autopsy with examination of organs as many social and religious implications in Kuwait hinder the following practice. Therefore at least a full external exam was performed in all accidental deaths in addition to toxicological screening. In only a some cases was an internal examination done with histopathological microscopic examination.

The data parameters that were analyzed were the association of type of accidental death with age, gender, nationality, marital status and governate.

The regional ethical review board of GDCE in the MOI of Kuwait approved the study provided the confidentiality of each case was maintained.

Data was analyzed using SPSS 17.0 (Chicago, USA). Statistical analyses involved computation of descriptive statistics for all demographic factors such as age and sex in the sample. Descriptive statistics were used to show the percentage of reported medico legal cases in different categories. Fisher’s exact test was used to find out the association between the different qualitative variables. A value of p < 0.05 was taken as significant.

## Results

Accidental deaths in Kuwait constituted 85.7% of all cases of un natural deaths.

The results from Table 1 show that RTAs were the leading cause of accidental deaths in Kuwait accounting for (3,156; 64.6%) of the total accidental cases. This was followed by death due to falls from heights which accounted for 639 (13.1%), followed by death due to drug overdose accounted for (303; 6.2%). The lowest reported cases were for deaths due to poisoning and they accounted for (50; 1%).

**Table 1:** **Total number, percentage and causes of accidental medico-legal death cases that were reported to the GDCE in Kuwait between 2003 and 2009**

**Type of accidental death**	**Number of cases (percentage)**
RTA	3156 (64.6)
Fall from height	639 (13.1)
Drug overdose	303 (6.2)
Burn	246 (5)
Asphyxia	136 (2.8)
Occupational Injury	124 (2.5)
Drowning	108 (2.2)
Electric shock	67 (1.4)
Mine explosion	57 (1.2)
Poison	50 (1 )

Table 2 shows that Non-Kuwaiti Residents had the highest incidence of mortality in all categories of accidental deaths except in cases of drug overdose were Kuwaitis were almost double the numbers of non-Kuwaitis. There was a significant difference p = 0.000 in all categories of accidental deaths classified according to nationalities comparing Non Kuwaiti residents with Kuwaiti.

**Table 2:** **Total number, percentage and causes of reported medico-legal cases of accidental deaths according to nationalities between 2003 and 2009**

**Type of accidental death**	**Number of Kuwaiti cases (percentage)**	**Number of non-Kuwaiti cases (percentage)**
RTA	1276 (40.5)	1876 (59.5)
Fall from height	96 (15)	543 (85)
Drug overdose	201 (66.8)	100 (33.2)
Burn	103 (41.9)	143 (58.1)
Asphyxia	62 (45.6)	74 (54.4)
Occupational Injury	10 (8.1)	114 (91.9)
Drowning	45 (42.9)	60 (57.1)
Electric shock	11 (16.4)	56 (83.6)
Mine explosion	3 (2.6)	54 (97.4)
Poison	21 (42)	29 (58)

The results in Table 3 show that male victims outnumbered female victims in all categories of accidental deaths. The results show a statistical significance (p < 0.000).

**Table 3:** **Total number, percentage and causes of medico-legal accidental death cases that were reported to the GDCE in Kuwait according to gender between 2003 and 2009**

**Type of accidental death**	**Number of Male cases (percentage)**	**Number of female cases (percentage)**
RTA	2636 (83.5)	520 (16.5)
Fall from height	523 (82.3)	116 (17.7)
Drug overdose	286 (94.4)	17 (5.6)
Burn	150 (61)	96 (39)
Asphyxia	86 (63.2)	50 (36.8)
Occupational Injury	107 (86.3)	17 (13.7)
Drowning	90 (84.9)	16 (15.1)
Electric shock	62 (92.5)	5 (7.5)
Mine explosion	55 (96.5)	2 (3.5)
Poison	44 (88)	6 (12)

Table 4 shows that more people died from the married group compared to the non-married (un-married or single) group due to RTA, burn injuries, drug overdose, death due to electrocution, falls from heights, mine explosions and occupational injuries. In contrast, in non-married group the number of deaths were higher than the married group in deaths due to asphyxia and drowning. No statistical significance in the difference was recorded.

**Table 4:** **Total number and causes of medico-legal cases of accidental deaths that were reported to the GDCE in Kuwait according to marital status between 2003 and 2009**

**Type of accidental death**	**Number of Married cases (percentage)**	**Number of non-married cases (percentage)**
RTA	1679 (53.3)	1471 (46.7)
Fall from height	332 (52.1)	305 (47.9)
Drug overdose	172 (56.8)	131 (43.2)
Burn	126 (51.2)	120 (48.8)
Asphyxia	54 (41.2)	77 (58.8)
Occupational Injury	65 (52.4)	59 (47.6)
Drowning	37 (34.3)	71 (65.7)
Electric shock	45 (67.2)	22 (32.8)
Mine explosion	40 (70.2)	17 (29.8)
Poison	25 (50)	25 (50)

In Table 5 we divided our case population into different age groups and calculated the incidence in each type of accidental deaths. The 20–29 and 30–39 age groups accounted for the highest number of deaths in RTA, fall from height, drug overdose, occupational injury, electric shock, mine explosion and poisoning, with p value <0.05. However, the 0–9 age group shows the highest incidence in deaths due to drowning, asphyxia (p < 0.05).

**Table 5:** **Total number (percentage) of the different types of accidental deaths according to different age groups**

**Type of accidental death**	**0-9**	**10-19**	**20-29**	**30-39**	**40-49**	**50-59**	**>60**
RTA	244 (7.7)	403 (12.8)	842 (26.7)	704 (22.3)	511 (16.2)	242 (7.7)	210 (6.6)
Fall from height	87 (16.1)	31 (4.8)	155 (27.2)	193 (29.2)	101 (13.3)	47 (6.4)	25 (3)
Drug Overdose	3 (1)	8 (2.5)	103 (34)	106 (35)	70 (23)	12 (4)	1 (0.5)
Burn	62 (25.2)	19 (7.7)	39 (15.9)	52 (21.1)	32 (13)	16 (6.5)	26 (10.6)
Asphyxia	49 (36)	11 (8)	19 (14)	22 (16.2)	19 (14)	6 (4.4)	10 (7.4)
Occupational Injury	0	3 (2.4)	50 (40.3)	42 (33.8)	20 (16.3)	9 (7.2)	0
Drowning	39 (36.1)	16 (14.8)	16 (14.8)	15 (13.9)	8 (7.4)	9 (8.3)	5 (4.6)
Electric shock	3 (4.5)	1 (1.5)	25 (37.3)	16 (23.9)	11 (16.4)	8 (11.9)	3 (4.5)
Mine explosion	0	1 (1.8)	24 (42.1)	20 (35.1)	9 (15.8)	2 (3.5)	1 (1.8)
Poison	0	1 (2)	20 (40)	21 (42)	7 (14)	1 (2)	0

Farwaniya governate had the highest number of accidental deaths in the study period. The least number of cases were in Mobarak Al-Kabeer governate (Table 6).

**Table 6:** **Numbers of accidental deaths in the different governates of the State of Kuwait**

**Governate**	**Number (percentage)**
Jahra	934 (19.1)
Farwania	1418 (29)
Hawally	739 (15.1)
Ahmady	930 (19.1)
Mobarak Al-Kabeer	239 (4.9)
Capital	626 (12.8)

Finally, the results of toxicological screening revealed that the highest number of accidental alcohol-related deaths were reported in the year 2006 accounting for (62; 17.97%) of the total number of accidental deaths in that year. The least number of alcohol-related deaths were reported in 2009 accounting for (38; 11.01%).

## Discussion

Kuwait has the world’s highest traffic accident death rate despite its small total population of around three million. The country is spending $95 billion USD in tackling the problem [3]. Children sit in the front seat, accounting for their high mortality. Road designs are critical in addressing the problem of traffic density and congestions. These are due to the lack of an effective traffic routing model in Kuwait. From our data alcohol consumption seems to have a non- significant impact on accidental deaths due RTA.

In accidental fall from heights the majority of the adult females were domestic caregivers instructed by the house owners to clean the windows on high floors. This kind of practice should be strictly prohibited by the legal authorities in Kuwait and certified companies should be the sole provider of this service. Children succumbed to these domestic accidents directly attributed to parents’ or caregivers’ negligence. Furthermore, a major cause of falls from heights was due to window cleaning at work, especially from large buildings [4] because the workers failed to observe simple safety precautions during climbing. A study was conducted in Kuwait University, Department of Civil Engineering, presenting an analysis of construction-related accidents in Kuwait along with the causes of accident injuries that were fatal. The research confirmed that construction was the most hazardous industry in Kuwait, with accidents accounting for 48% of work- related-deaths in 1996 [5], while in the U.S.A. it accounted for only 14% of all work-related deaths in the same year [6].

Males outnumbered females in all categories of accidental deaths. The differences may be due to the lack of attention and negligence of safety protocols among males that constitute the bulk of laborers in Kuwait. It can also reflect the lack of education provided to laborers about safety in the workplace.

The highest number of fire deaths was among children. Kuwait is highly dependent on house keepers and baby sitters. Most of the caregivers are uneducated and they often possess limited knowledge in dealing with fire and fire-related incidences. Moreover, the employers does not provide necessary training in dealing with fire hazards, neither is there a national program to educate people about this issue.

Deaths due to drug overdose were the third most common cause of accidental deaths. Victims are mostly young Kuwaiti males despite the steady efforts made by the MOI to combat drug trafficking at its source. Family awareness is needed, followed by community alertness to eliminate bad habits in order to save lives. Strict law and religious doctrine in Kuwait forbid drugs, providing a significant cultural deterrent. Together, these may lead to a decrease in the rate of drug abuse in Kuwait.

Most deaths due to accidental drowning involved accidents in swimming pools, bathing tubs, boating or other water sporting activities. About 72% of the houses in Kuwait have a built-in swimming pool either indoor or outdoor [7].Children are left to swim alone without proper supervision. In 2010 a UNICEF report estimated almost half a million deaths worldwide are caused by drowning [8]. It was the second leading cause of injury-related deaths with 57% of these being children up to 14 years of age. Moreover, in adults drowning is related to fishing incidents. The latter is a common hobby among many youth in Kuwait for centuries since Kuwait is at the Arabian Peninsula, which is a big waterfront. However many of these youth buy a boat and go fishing without the presence of proper precautions such as floating devices and some of them do not know how to swim. The government needs to take strict precautions before allowing people to buy a boat or go fishing. Campaigns need to be in place to educate the public about safety precautions in the sea.

In Kuwait poisons are not commonly found in households and workplaces and this accounts for the low number of deaths.

Deaths due to electrocution were mostly men who had little or no professional training in handling electricity and in simple safety procedures. These accidents and deaths occurred mainly at homes and also at work especially in the electrical construction occupation. Construction workers are still about four times more likely to be electrocuted at work than workers in all the other industries combined [9-12]. However, the domestic electrocutions represent a huge gap in safety education that needs to be filled by the official authorities in Kuwait.

Deaths due to mine explosion showed a significant (p < 0.000) difference in males compared to females. These are mostly a direct result of Iraqi invasion to Kuwait in 1990. The Iraqi regime brought massive quantities of army materials into Kuwait. Since liberation until a few years ago, about seven million landmines have been identified in Kuwait placed by the Iraqi regime. Casualties in Kuwait since that period reached hundreds of cases of both civilians and military personnel. Especially in the north where there are many farms and many shepherds (mainly expatriates) that take their sheep around the farms. Cases of mine explosions are widespread in countries that suffer from war. Afghanistan, Bosnia, Cambodia, and Mozambique have suffered economic, social and human lossess due to mine explosions [13].

In our study, several forms of asphyxia were combined because of the small number of cases. These include suffocation, choking, positional asphyxia/wedging in children and suffocative asphyxia due to different gases and other accidental types. The increase in the number of reported cases in the very young age group may be due to the fact that young children are at an increased risk and more vulnerable to asphyxia. Similar findings have been reported in USA [14]. Young children are particularly vulnerable to asphyxia due to choking on food because of their small and immature airway and their inability to bite and chew food [15,16]. About 14 cases of accidental deaths due to asphyxia resulting from gas poisoning (phosphine) were reported. The basements in these houses were rented to companies that used the basement as a store for commercial items. The companies also used insecticide to prevent insects from penetrating there products. As a result, phosphine gas could spread easily from the basement to flats and floors above where the families lived. Following these reported incidents, the Ministry of Planning in Kuwait banned the use of basements for the storage of insecticides.

## Conclusion

Accidental deaths in Kuwait pose a public health problem. It seems that education is the most important factor in reducing those kinds of deaths. The government of Kuwait, as part of its strategic plan, needs to secure funding for education and public awareness of workplace and home related hazards. More strict enforcement of roadside rules would be the best solution to the high RTA in Kuwait.

## Abbreviations

RTA: Road traffic accidents
